# Arthroscopic Lower Trapezius Tendon Transfer for a Patient with Axillary Nerve Injury and Concomitant Rotator Cuff Tear: A Case Report and Technical Notes

**DOI:** 10.3390/medicina59101817

**Published:** 2023-10-12

**Authors:** Jeff Loren, Chuieng-Yi (Johnny) Lu, Cheng-Pang Yang, Kuo-Yao Hsu, You-Hung Cheng, Huan Sheu, Chao-Yu Chen, Hao-Che Tang, Chieh-An Chuang, Chih-Hao (Joe) Chiu

**Affiliations:** 1 Department of Orthopedic Surgery, Linkou Chang Gung Memorial Hospital, Taoyuan 333, Taiwan; jeffloren@gmail.com (J.L.); ronnie80097@gmail.com (C.-P.Y.); alvinchen@cgmh.org.tw (C.-Y.C.); 2 Department of Orthopedics & Traumatology, Royal Prima General Hospital, Medan 20118, Indonesia; 3 Division of Reconstructive Microsurgery, Department of Plastic and Reconstructive Surgery, Chang Gung Memorial Hospital, Taoyuan 333, Taiwan; cylu122@gmail.com; 4 Department of Occupational Therapy and Graduate Institute of Behavioral Sciences, College of Medicine, Chang Gung University, Taoyuan 333, Taiwan; 5 Department of Orthopedic Surgery, New Taipei Municipal Tucheng Hospital, New Taipei City 236, Taiwan; emsequoia@gmail.com (K.-Y.H.); ckbaboon@gmail.com (Y.-H.C.); 6 Department of Orthopedic Surgery, Taoyuan Chang Gung Memorial Hospital, Taoyuan 333, Taiwan; wondergillian@gmail.com; 7 Department of Orthopedic Surgery, Keelung Chang Gung Memorial Hospital, Keelung 204, Taiwan; tanghaoche@gmail.com (H.-C.T.); power_of_kevin@hotmail.com (C.-A.C.)

**Keywords:** brachial plexus injury, axillary nerve injury, lower trapezius tendon transfer, Achilles allograft

## Abstract

*Introduction*: Concomitant nerve injuries with musculoskeletal injuries present a challenging problem. The goals of nerve reconstruction for the shoulder include shoulder abduction and external rotation. When patients fail to achieve acceptable shoulder external rotation and shoulder abduction, tendon transfers such as trapezius transfer offer a reliable option in the subsequent stage. *Case Presentation*: A 32-year-old male presented with weak external rotation in his left shoulder, after previous axillary nerve reconstruction. He received the ipsilateral lower trapezius transfer with the aim of improving the external rotation. *Discussion*: The lower trapezius restores a better joint reaction force in both the compressive–distractive and anterior–posterior balancing and provides a centering force through the restoration of the anterior–posterior force couple. *Conclusion*: We believe that the ipsilateral lower trapezius transfer to the infraspinatus is a good outcome and is effective in improving overall shoulder stability and the shoulder external rotation moment arm or at least maintaining in neutral position with the arm fully adducted in patients with post axillary nerve injuries post unsatisfactory nerve reconstruction to increase the quality of life and activities of daily living.

## 1. Introduction

Traumatic brachial plexus injuries (BPIs) are devastating, life-altering injuries that result in significant physical disability, psychological distress, and socioeconomic problems [1,2]. These injuries most commonly occur in men, with an average age of 28.38 years [3]. A lack of shoulder function after a traumatic BPI can be attributed to either (1) the suprascapular nerve, which innervates the supraspinatus and infraspinatus muscles, or/and (2) axillary nerve injuries, which innervate the deltoid and teres minor muscles. A C5–C6 root avulsion injury can lead to a decrease in abduction strength by 75% and a decrease in external rotation strength by 80% [4,5,6]. Reinnervation of the aforementioned muscles can be achieved after nerve reconstruction, but glenohumeral joint stability and mobility are prerequisites to achieving acceptable outcomes [4,7]. Isolated axillary nerve injury patients are technically not BPIs, but their proximity to the cords in the infraclavicular region and potential injury of the surrounding musculoskeletal structures renders its outcomes unpredictable. If the external rotator muscles fail to become innervated, the patient shows an internally rotated shoulder position (‘‘hand on belly’’) and a limitation of the functional use of the upper extremity in many activities of daily living (ADLs) [8,9,10,11].

When outcomes after nerve reconstruction are unsatisfactory, or a shoulder joint-related injury remains unresolved, secondary surgeries can restore external rotation function or at least decrease the degree of the internally rotated shoulder position, thereby enhancing shoulder function [7]. Tendon transfers, such as trapezius or latissimus dorsi transfers, or shoulder arthrodesis with intact scapulothoracic muscles can help stabilize the shoulder and potentially improve external rotation [6,12,13,14].

The trapezius has three functional segments: a descending segment that supports the weight of the arm; a transverse segment that retracts the scapula; and an ascending segment that medially rotates and depresses the scapula [14]. Transfers of the lower trapezius tendon (LTT) with Achilles allograft have been proven to improve shoulder external rotation in patients with massive, irreparable posterosuperior rotator cuff tears [10,15,16,17,18,19]. We operated on three patients with a loss of axillary nerve function and shoulder external rotation. One distinctive characteristic of a patient within this group is that he had a concomitant rotator cuff tear. This particular circumstance encouraged us to use an Achilles allograft in conjunction with an LTT transfer. For patients without a rotator cuff tear, we aim to enhance the shoulder external rotation using the Inlay technique [2] with an autologous hamstring tendon. The purpose of this technical note is to describe the procedure of arthroscopic-assisted LTT transfer with Achilles allograft in a patient with axillary nerve disruption following shoulder dislocation and a rotator cuff tear. He had undergone a shoulder dislocation rehab regimen for 8+ months prior to nerve exploration which delayed his timing. The procedure aims to improve his external rotation.

## 2. Clinical Cases

### 2.1. Case

**Figure 1:**
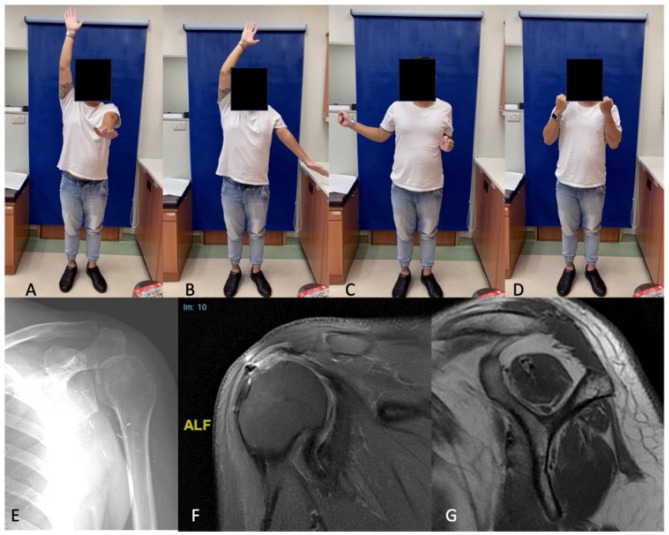
Preoperative physical and image findings of the patient. (**A**) The patient could only forward elevate the shoulder to 80° and (**B**) laterally abduct to 45°, with (**C**) an external rotation by the side of about 0°. (**D**) Elbow and wrist flexion were maintained. (**E**) X-ray of the left shoulder revealed a malunited proximal humerus. (**F**,**G**) MRI revealed a rotator cuff tear with Goutallier grade 1 muscle fatty infiltration.

A 32-year-old male dislocated his left shoulder in a devastating traffic accident, which necessitated a cuff repair. His shoulder abduction and external rotation remain paralyzed after eight months of rehabilitation, and electrodiagnostic studies indicated a posterior cord injury. Exploration of the supraclavicular plexus showed an intact C5–C6 root, with stimulation of the suprascapular nerve eliciting muscle contractions. However, exploration of the infraclavicular region showed a completely disrupted axillary nerve, with a gap of about 10 cm. Axillary nerve grafting with neurolysis of the radial nerve was performed at ten months after the initial injury, and after one year of post-operative rehabilitation, he could only actively forward elevate the shoulder to 80° and laterally abduct to 45°, with an external rotation by the side of about 0° (Figure 1A–C). His elbow and wrist flexion were maintained (Figure 1D), but the internally rotated shoulder position (hand on belly) was markedly evident. A standard X-ray of the left shoulder revealed a malunited proximal humerus (Figure 1E). An MRI revealed a rotator cuff tear with Goutallier grade 1 muscle fatty infiltration (Figure 1F,G).

Patient Indication:

The indication and contraindication of LTT transfer for traumatic BPIs is shown in Table 1 [6,10,14,20].

### 2.2. Surgical Technique

#### 2.2.1. Patient Preparation

**Figure 2:**
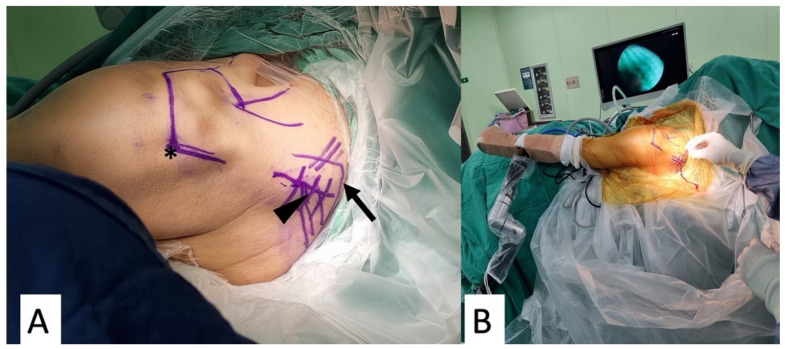
The patient was put in a beach-chair position with an arm traction device. (**A**) Left shoulder, viewing from back. Arrow, medial border of the scapula. Arrowhead, scapular spine. Asterisk, posterolateral border of the acromion. (**B**) The posterior shoulder was better approached with a traction device.

The patient was placed in a beach-chair position under general anesthesia with an interscalene block. The medial scapular border should be easily accessible. We marked the medial border of the scapula, the scapular spine, and the posterolateral border of the acromion for a better approach (Figure 2A). The operated arm was placed into a hydraulic arm holder (DJO, ENOVIS, Carlsbad, CA, USA) (Figure 2B).

#### 2.2.2. Graft Preparation

##### Lower Trapezius Tendon Harvest and Preparation

**Figure 3:**
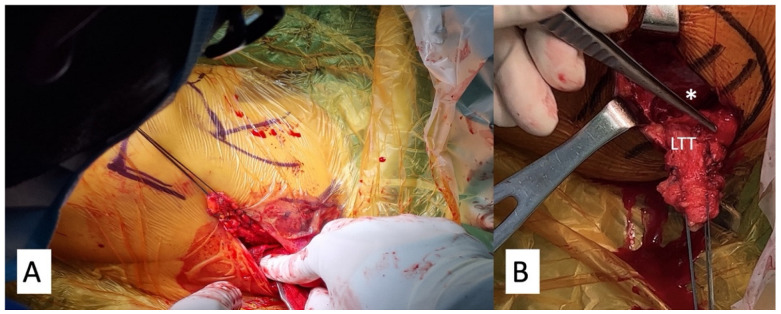
Lower trapezius tendon harvest. (**A**) The tendon was sutured with 2 sets of No. 2 Ethibond sutures. View from the back. (**B**) The tendon was separated from the infraspinatus muscle (*) in the deeper layer. LTT, lower trapezius tendon.

The skin incision was made slightly horizontal (transverse), starting from the medial third inferior portion of the scapula spine and extending to the medial edge of the scapula. Instead of the technique proposed by Bassem et al. [21], which first exposes the adipose tissues inferior to the LTT, we opened the fascia of the LTT directly at its insertion on the scapular spine using electrocautery. After separating the interval horizontally between the upper trapezius and lower trapezius from the scapular spine, a vertical cut was performed in the middle of the scapular spine, exposing the lateral most part of the LTT. Blunt dissection was then performed to separate the LTT and the infraspinatus muscle in the deeper layer, until reaching the medial border of the scapula. The lateral stump of the LTT was sutured with two pairs of the No. 2 Ethibond suture (Ethicon, Somerville, NJ, USA) in a Krackow stitch manner (Figure 3A,B).

##### Achilles Allograft Tendon Preparation

**Figure 4:**
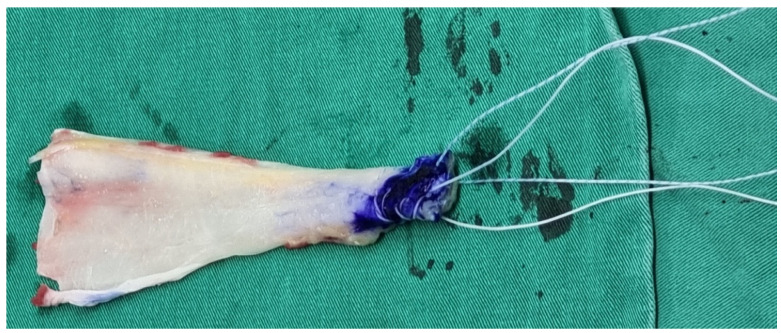
The calcaneus part of the Achilles allograft was sutured with two sets of Ultrabraid sutures.

An Achilles allograft without the bony part was used. The calcaneus part was sutured with two sets of Ultrabraid sutures in a Krackow stitch manner (Figure 4). The smoother part served as the articular side and the rougher part as the bursal side when introduced into the shoulder joint.

#### 2.2.3. Arthroscopic Achilles Allograft Tendon Passage and Fixation

**Figure 5:**
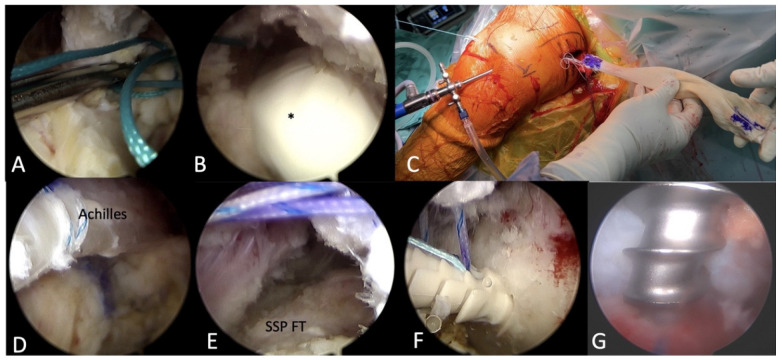
Arthroscopic Achilles Allograft Tendon Passage and Fixation. (**A**) A grasp with one No. 5 Ethibond suture was shuttled above the infraspinatus muscle and beneath the scapular spine, through the infraspinatus muscle fascia. (**B**) A blunt dissection with a finger (*) was performed to clear the space facilitating the passage of the Achilles allograft. (**C**–**E**) The Achilles allograft was pulled intra-articularly until it covered the footprint of the anterior part of the supraspinatus. (**F**) A lateral row anchor was used to preliminarily fix the graft. (**G**) A double-loaded anchor was inserted into the infraspinatus footprint, facilitating the fixation between Achilles allograft and infraspinatus tendon. SSP FT, supraspinatus footprint.

Posterior, anterior, anterolateral, and lateral portals were created using a stabbing incision. A 4 mm lens with a 30-degree Arthroscopy was used with a fluid pump pressure at 30 mmHg. The native footprints of the supraspinatus and infraspinatus were identified and debrided until a bleeding surface was exposed. Viewing from the lateral portal, the scapular spine was identified intra-articularly as the interval between the supraspinatus and infraspinatus. A grasp with one No. 5 Ethibond suture was shuttled above the infraspinatus muscle and beneath the scapular spine, through the infraspinatus muscle fascia, until it penetrated into the wound where we harvested the LTT at the posterior shoulder (Figure 5A). Then, a blunt dissection with a finger was performed to clear the space along the Ethibond suture, facilitating the passage of the Achilles allograft (Figure 5B).

The Ethibond suture outside the posterior wound was tied with two sets of sutures from the Achilles allograft, and it was pulled intra-articularly with the other end of the Ethibond suture (Figure 5C,D) until it covered the footprint of the most anterior part of the supraspinatus (Figure 5E). Two lateral row anchors (5.5 mm Footprint PK, Smith & Nephew, Andover, MA, USA) were used to preliminarily fix the graft (Figure 5F). One anchor was placed at the bicipital groove and the other at the greater tuberosity. Then, two other double-loaded anchors (Twinfix Ti 5.0, Smith & Nephew, Andover, MA, USA) were inserted into the middle of the supraspinatus footprint and the infraspinatus footprint (Figure 5G). Two sets of mattress sutures were passed through the Achilles allograft from the anterior anchor to fix it on the supraspinatus footprint. Then, one limb of the suture from the posterior anchor was passed through the Achilles allograft, and the other limb through the infraspinatus, to work as a mattress suture. The two sets of mattress sutures from the posterior anchor fixed both the Achilles allograft and infraspinatus onto the infraspinatus footprint.

#### 2.2.4. Merging the Achilles Allograft with LTT

**Figure 6:**
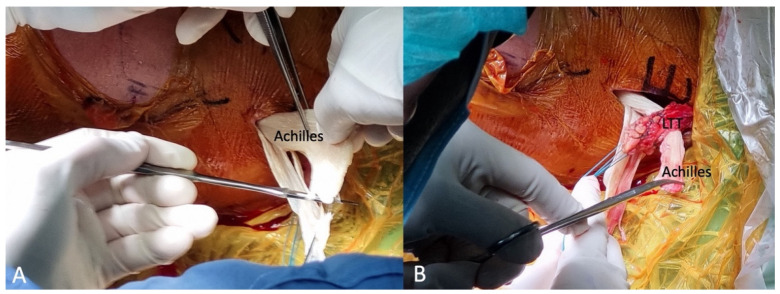
Merging the Achilles allograft with the lower trapezius tendon. (**A**) The fascia part of the Achilles allograft was sectioned in half to decrease the bulky side. (**B**) The merging of LTT and Achilles was performed using multiple No. 2 Ethibond sutures with Pulvertaft weaving at one end of the Achilles allograft. LTT, lower trapezius tendon.

The LTT was then sutured together with the Achilles allograft while the operated shoulder was placed in maximal abduction and external rotation to compensate for the internally rotated “hand-on-belly” position. The fascia part of the Achilles allograft was sectioned in half to decrease the bulky side (Figure 6A). The merging was performed using multiple No. 2 Ethibond sutures with Pulvertaft weaving at one end of the Achilles allograft (Figure 6B). The operative wound was then closed in the usual fashion.

### 2.3. Postoperative Protocol

**Figure 7:**
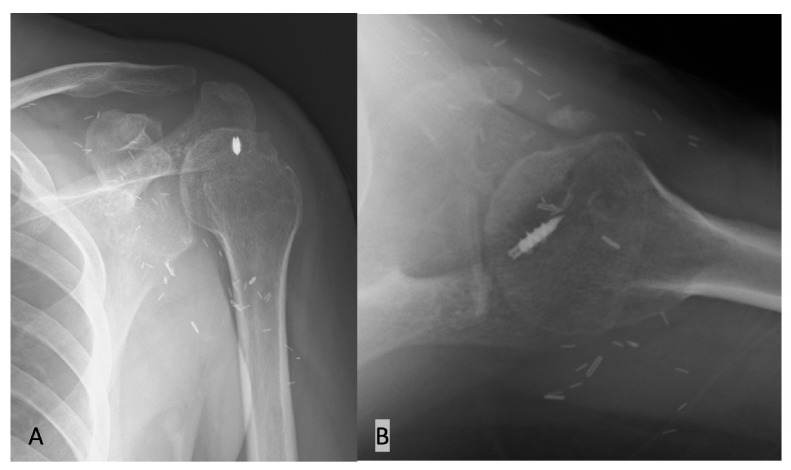
X-ray follow ups at 3 months after surgery. (**A**) Anteroposterior view. (**B**) Axillary view.

Post-operation, the patient was fitted with a shoulder abduction external rotation brace in 30° of abduction and 30° of external rotation for 24 h a day, which limited their range of motion for six weeks. From six to twelve weeks, the brace was removed, and the patient began both pain-free passive and active exercises in elevation and external rotation with a night splint to keep the hand away from the belly. Three months post-operation, patients are encouraged to be independent and to engage in an active-assisted range of motion for their activities of daily living (ADLs) with gentle strengthening. An X-ray was taken right after surgery and at a 3-month follow up (Figure 7). Water-based therapy is recommended at this time, along with stretching in internal rotation. No limitations on activities are needed after 6 months, although strength will continue to increase for 12 or even 18 months [22].

## 3. Discussion

The shoulder joint achieves balance in both vertical and horizontal planes, thanks to the counterbalance provided by each muscle [4,23]. The rotator cuff muscles primarily serve as dynamic stabilizers for the shoulder during abduction, forward flexion, and extension. They resist the upward shearing moment exerted by the deltoid at the glenohumeral joint during early abduction, facilitated through a force couple involving the subscapularis anteriorly and the infraspinatus posteriorly [4,24].

Following a peripheral nerve injury, the shoulder loses its balance in force couples, resulting in inferior shoulder subluxation. This imbalance is due to the loss of dynamic stabilizers in the glenohumeral joint, causing the humeral head to become uncontained. Consequently, this can lead to pain due to glenohumeral subluxation [4,16,25].

The trapezius is a substantial muscle, boasting an excursion of 13 cm and a relative tension of 12.4%. In comparison, the infraspinatus has an excursion of 8.6 cm and a relative tension of 9.7% [25]. The lower trapezius possesses a force vector that aligns with the direction of the infraspinatus, making it an optimal choice for tendon transfers [26,27]. Utilizing the LTT adheres to the principles of tendon transfers, which encompass similar excursion and tension, expendability, a parallel line of pull, and the replacement of only one function of the recipient muscle [28,29].

In biomechanical studies comparing the external rotation moment arm between the latissimus dorsi (LD), teres major, and LTT, the LTT transfer demonstrates the most substantial moment arm for external rotation when the shoulder is fully adducted (0° abduction). Conversely, the LD transfer exhibits the smallest moment arm in this position [11]. However, this dynamic changes when the shoulder is abducted at 90°. In this position, the LD boasts the largest moment arm for external rotation, while the lower trapezius has the least significance [30,31]. However, LD transfer, indicated in children suffering from obstetric brachial plexus palsy (OBPP) to restore forward elevation and external rotation, has no role in adults of traumatic brachial plexus palsy. This muscle is often paralyzed due to long thoracic nerve damage, and its action causes inferior subluxation of the humeral head, given the paralysis of the deltoid and rotator cuff muscles [1]. Regarding utilizing the upper trapezius to restore shoulder ROM, the results have always been very modest with abductions and no external rotations. This transfer harvests an acromion fragment and more anterior fixation on the humerus to restore some forward elevation but not external rotation [2,3]. Lastly, humeral de-rotation osteotomy is the ultimate palliative technique to prevent rubbing of the arm on the thorax, but it does not allow for active external rotation. This technique is commonly used for sequelae of OBPP and may be indicated if the shoulder joint is stiff or arthritic and destroyed [4] but not for patients with external rotation loss. In summary, LTT transfer offers a superior joint reaction force in both compressive–distractive and anterior–posterior balancing. It also provides a centering force through the restoration of the anterior–posterior force couple [32].

We opt for the LTT transfer based on the several biomechanical advantages it offers. Its cranial origin placement results in a reduced humeral head depression, and its line of pull closely resembles that of the infraspinatus. Additionally, the LTT transfer shares similar excursion and tension characteristics with the infraspinatus [17,19]. Comparative studies suggest that the LTT transfer better restores shoulder kinematics and glenohumeral force couples in an adducted position than other tendon transfers [17,33]. While the joint reaction forces post-LTT transfer might not match those of a normal shoulder, this is not a significant concern. This is primarily because the shoulders of BPI patients are predominantly in the adduction position. Our approach to treating patients with traumatic BPI is a modification of techniques published by Elhassan et al. and Chiu et al. [21,34].

## 4. Conclusions

For patients suffering from BPI or axillary nerve injuries with a paralytic shoulder, who predominantly keep their arm by their side, the LTT transfer presents an effective solution to restore shoulder external rotation. By adopting this surgical approach, not only can their quality of life be significantly enhanced, but their capability to carry out daily activities can also see a marked improvement.

## Figures and Tables

**Table 1:** The indication and contraindication of LTT transfer for traumatic BPIs.

Indication	Contraindication
- Intra-plexus Nerve Injury - Flail shoulder in the setting of traumatic Brachial Plexus Injury (BPI) - Trapezius muscle have a full strength against resistance based on Modified British Medical Research Council Scale (4/5 or 5/5) - Preoperative passive shoulder abduction is at least 80°	- Extra-plexus (Spinal accessory) nerve has been used for nerve transfer to the suprascapular nerve - Spinal accessory nerve has been used as donor in free functional muscle transfer (FFMT). - Patient with cervical injury (C3, C4) - Active soft tissue infection Glenohumeral degenerative changes - Inability to follow postoperative recommendations

## Data Availability

Not applicable.
